# Iodine-Induced Hypothyroidism After Chemoembolization With Ethiodized Oil: A Case of Failure to Escape From Wolff-Chaikoff Effect (WCE)

**DOI:** 10.7759/cureus.39352

**Published:** 2023-05-22

**Authors:** Ana de Carmo Campos, Isabel Cruz Carvalho, Sara Sarmento, Teresa Fonseca

**Affiliations:** 1 Pulido Valente Hospital, North Lisbon University Hospital Centre (CHULN), Lisbon, PRT; 2 Health Promotion and Prevention of Non-Communicable Disease, National Health Institute Doutor Ricardo Jorge, Lisbon, PRT; 3 Medicine, Lisbon University, Lisbon, PRT

**Keywords:** drug adverse event, ethiodized oil, iodinated contrast media, hypothyroidism, thyroid dysfunction, wolff-chaikoff effect

## Abstract

Wolff-Chaikoff effect (WCE) is an acute physiologic response of the thyroid gland to high levels of iodine. The WCE is usually temporary, and the thyroid gland adjusts to high iodine levels within days or weeks. It is a protective mechanism and a failure to escape from it can result in thyroid dysfunction. Hypothyroidism is rare but more likely in patients who have had thyroid disease, are under stress, or are exposed to high iodine concentrations for a long time. The use of iodinated contrast media in radiologic studies or invasive medical procedures is a common source of excessive iodine exposure and can lead to thyroid disfunction. Despite the frequent use of contrast media in health care, the thyroid dysfunction associated with their use is poorly recognized.

We report a patient who developed iodine-induced hypothyroidism after chemoembolization of recurrent chylothorax with ethiodized oil (Lipiodol®, Villepinte, France). This case report raises awareness of the importance of the physiologic WCE as well as the relevance of monitoring thyroid function when using iodinated contrast media in patients with specific risk factors.

## Introduction

The WCE is an acute autoregulatory response to high levels of iodine [1]. It is a protective mechanism that temporarily reduces the production of thyroid hormones in response to high iodine intake, preventing a hyperthyroid state. [1-2]. Severe hyperthyroidism or thyroid storm treatment with iodine is based on this physiological principle and is one of the strategies to promote a patient’s return to a euthyroid state [2-3]. The WCE is usually self-limiting and after a few days to weeks, thyroid glands adapt to prolonged elevated levels of iodine and patients usually return to euthyroid state [4-5].

In rare cases, thyroid dysfunction (TD) can occur, especially in patients with risk factors [4]. The use of iodinated contrast media (ICM) in radiologic studies and interventional procedures is a common source of excessive iodine exposure in many patients and can lead to TD. Despite the frequent use of ICM in health care, the TD associated with their use is poorly recognized.

We report a patient who developed iodine-induced hypothyroidism after chemoembolization of recurrent chylothorax with ethiodized oil (Lipiodol®, Guerbert, Villepinte, France), as an absence of escape from WCE.

## Case presentation

**Figure 1:**
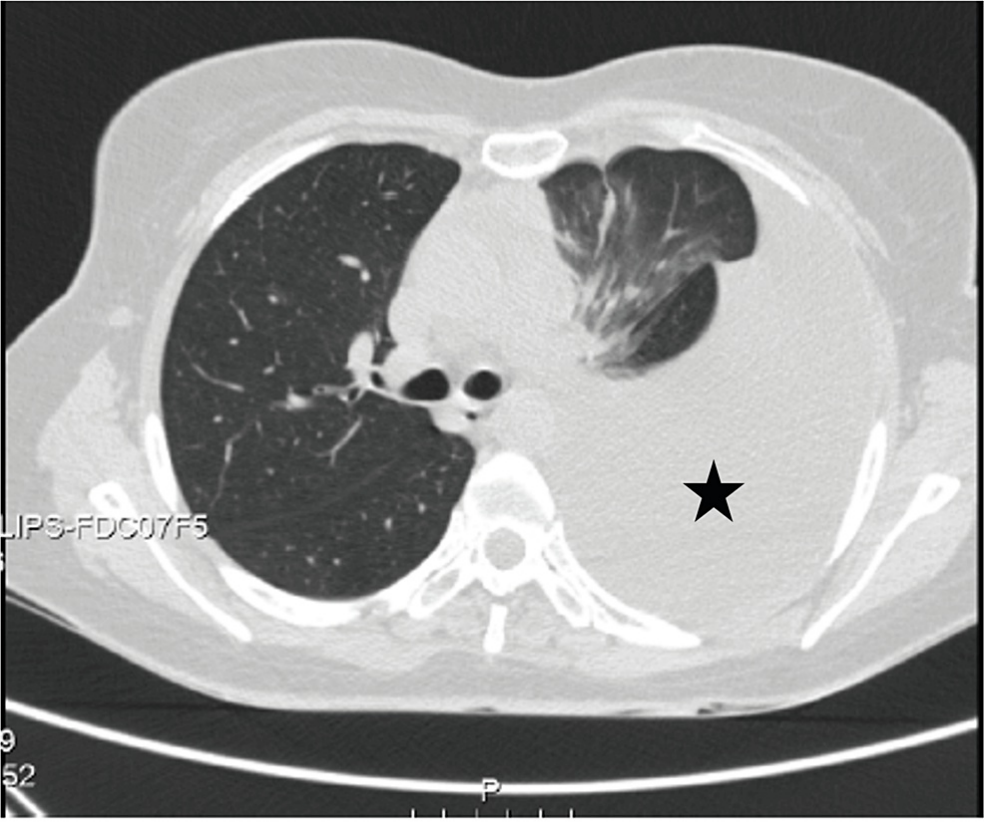
Left pleural effusion (chylothorax) (star) (CT axial lung window).

**Figure 2:**
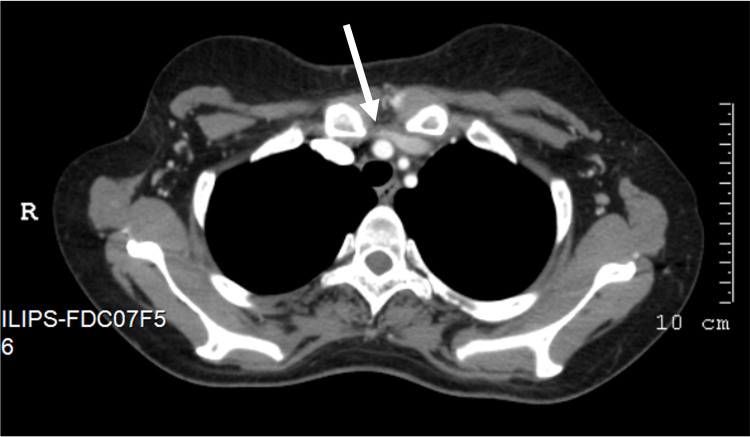
Stenosis of the left brachiocephalic venous trunk and thoracic duct (arrow) associated with adenopathies (CT chest axial plane).

A 27-year-old woman without previous thyroid dysfunction was admitted for fever and pancytopenia. The patient was clinically and biochemically euthyroid on admission [thyroid-stimulating hormone (TSH) 2.20 uU/mL, normal range 0.30-4.20 uU/mL; free thyroxine (FT4) 1.48 ng/dL, normal range 0.93-1.70 ng/dL]. Five days after, she became critically ill with persistent pancytopenia (hemoglobin 7.0 g/dL, normal range 12.0-15.3 g/dL; leukocytes 2.5 x 10^9^/L, normal range 4.0-11.0 x 10^9^/L; zero neutrophils, normal range 1.9-7.5 x 10^9^/L; platelets 91 x 10^9^/L, normal range 150-450 x 10^9^/L) and fever (maximum temperature has been 40ºC), with no response to antimicrobials, antivirals, tuberculostatic drugs and granulocyte colony-stimulating factor, in need of recurrent red blood cell and platelet transfusions. After an extensive study (namely, blood tests, blood culture tests, whole body CT, bone marrow aspiration and biopsy, lumbar puncture, lung biopsy, mediastinal lymph node biopsy, skin biopsy), the diagnosis of hemophagocytic lymphohistiocytosis (HLH) syndrome secondary to granulomatosis disease with pulmonary and extrapulmonary involvement was established. The patient was treated with corticosteroids and methotrexate. This clinical presentation was associated with multiple mediastinal adenopathies and complicated with left pleural effusion (Figure 1). After 600 mL of chylous fluid drainage with positive laboratory chylous test, chylothorax was diagnosed in relation to non-iatrogenic rupture of the thoracic duct (Figure 2).

**Figure 3:**
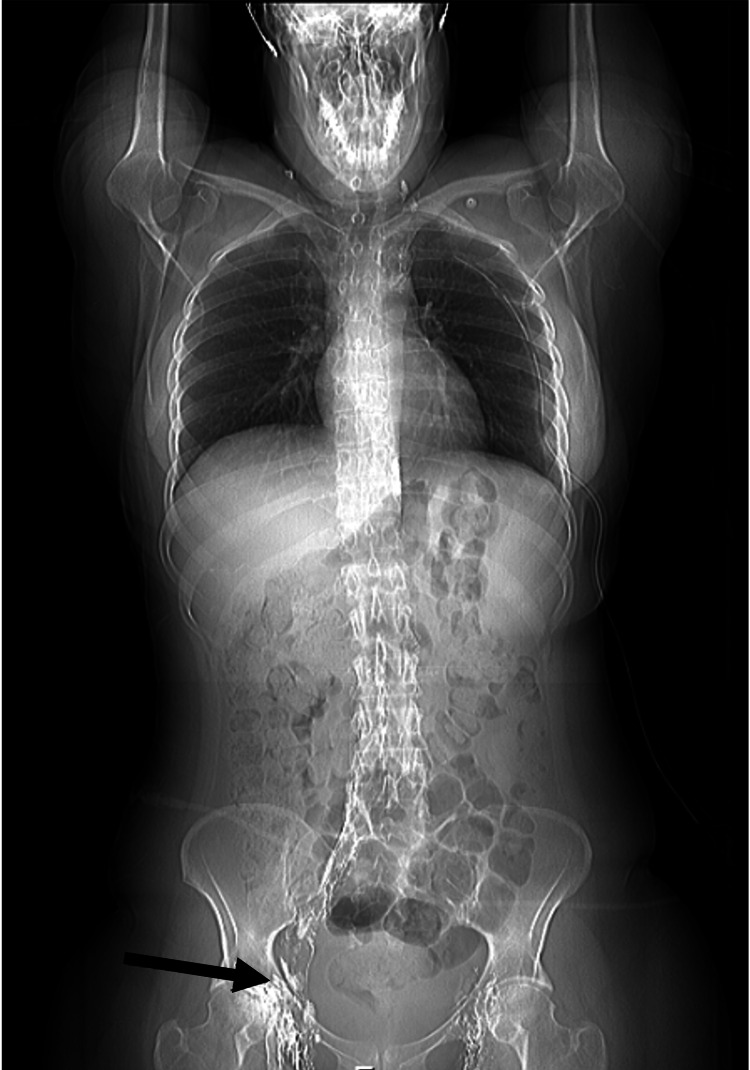
Radiographic image during intranodal lymphangiography with ultrasound-guided right inguinal lymph node approach to ethiodized oil injection (arrow).

**Figure 4:**
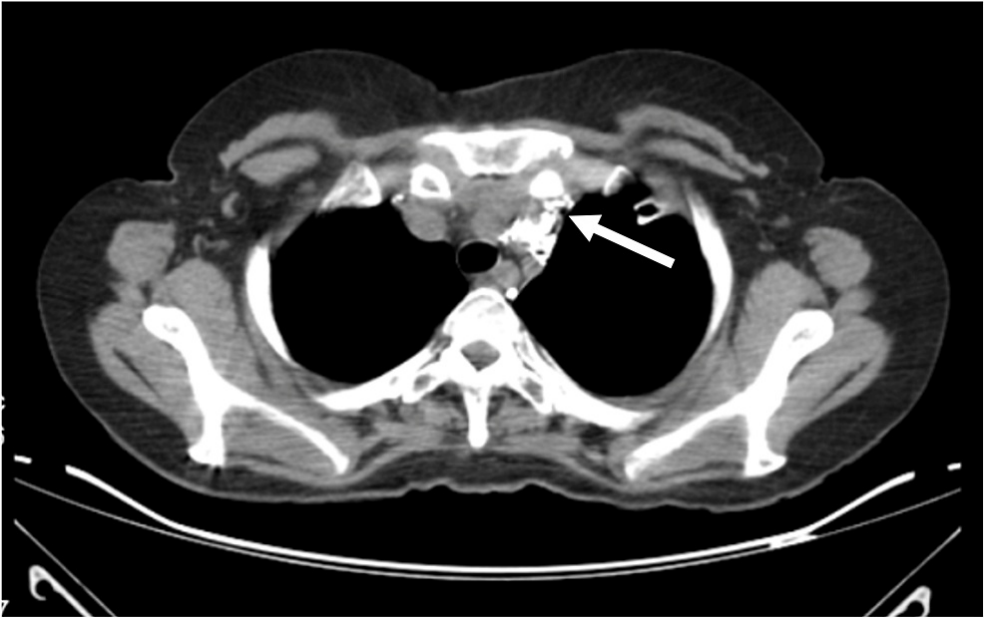
Thoracic duct embolization with Lipiodol®. Lymphatic opacification up to the union of the thoracic duct with the left subclavian vein, with leakage at left thoracic duct level (arrow) (CT chest axial plane).

The patient underwent conservative treatment for 10 weeks with persistent thoracic drainage (passive daily drainage of 70-250 mL in Atrium Express Mini 500® device, Atrium Medical Corporation, Hudson, NH), fatty food restriction, and octreotide administration, but it was ineffective. The patient underwent pleural talcage by left video-assisted thoracoscopic surgery (VATS), without chylothorax resolution. Recurrent chylothorax, persisting for four months, led to a multidisciplinary decision of thoracic duct chemoembolization and the patient received Lipiodol®-based intranodal lymphangiography. The thoracic duct was approached by ultrasound-guided percutaneous inguinal lymph node lymphangiography (Figure 3), and chylous leakage was identified and embolized using a total dose of 10 mL of Lipiodol® (0.1 mL/min) (Figure 4). The procedure was uneventful and chest drainage decreased to less than 30 mL/day three days after. A control image was achieved three days after lymphangiography and no leak was identified at the thoracic duct. The chylothorax resolved itself, the chest drain was removed, and the patient was discharged.

About one month after the lymphangiography, the patient started a clinical presentation suggestive of a hypometabolic state. The patient reported worsening fatigue, muscle weakness, hair loss, dry skin, obstipation, and cold intolerance.

On physical examination, her pulse was 76 beats per minute and her blood pressure was normal. She was well-nourished and hydrated. Her skin was dry, and her hair were scarce. There was no tremor observed. There was neither ophthalmopathy nor extraocular muscle weakness. A cardiovascular examination revealed normal heart sounds and no murmurs were found. The rest of the examination was unremarkable.

Laboratory test results were consistent with primary hypothyroidism (TSH 11.0 uU/mL, normal range 0.30-4.20 uU/mL; FT4 0.87 ng/dL, normal range 0.93-1.70 ng/mL) (Table 1). Thyroid peroxidase antibodies, thyroglobulin antibodies, and thyroid-stimulating immunoglobulin were negative. Ultrasonography of the thyroid gland showed no alterations. Hypothalamic pituitary axis was unchanged.

**Table 1:** Thyroid function tests before and after exposure to ethiodized oil. EO, ethiodized oil; TSH, thyroid-stimulating hormone; FT4, free thyroxine; FT3, free triiodothyronine; L-thyroxine, levothyroxine

Timeline of thyroid function	TSH (uU/mL)	FT4 (ng/dL)	FT3 (pg/mL)	L-thyroxine (μg/day)
Normal range	0.30-4.20	0.93-1.70	2.0-4.4	
At admission	2.20	1.48	2.14	-
One month before EO	2.52	1.04	-	-
Two weeks after EO (Day 14)	4.88	0.94	1.69	-
Five weeks after EO (Day 35)	7.26	0.93	-	-
Six weeks after EO (Day 42)	11.0	0.87	-	75
Eight weeks after EO (Day 56)	13.0	0.85	2.50	75
Nine weeks after EO (Day 69)	38.4	0.88	-	75
Three months after EO (Day 91)	4.30	1.56	1.99	75
Four months after EO (Day 120)	0.96	1.70	-	75
Seven months after EO (Day 200)	2.20	1.48	2.14	75
Eleven months after EO (Day 334)	0.87	1.68	-	50
Twenty-one months after EO (Day 652)	2.24	1.33	-	50
Two years after	3.08	1.68	3.27	-

The patient thyroid function was closely monitored for several weeks (Table 1), and the observed TD was assumed as iodine-induced hypothyroidism associated with the ICM in the embolized thoracic duct. Oral levothyroxine was initiated in a daily dose of 75 μg and adjusted to 50 μg daily after 11 months (Table 1). An euthyroid state was reached four months after exposure to ethiodized oil, and under thyroid hormone reposition for two months. About 20 months after exposure to ethiodized oil, the patient became pregnant and thyroid hormone replacement was maintained for another nine months. After delivery, her thyroid function remained normal, and treatment with levothyroxine was discontinued. The patient maintains annual monitoring of thyroid function with no changes to date.

## Discussion

The WCE was first described by Dr. Jan Wolff and Dr. Israel Chaikoff who investigated the effects of iodine on thyroid glands in the 1940s [2]. This mechanism is still not well understood but could be explained by the inhibition of thyroid peroxidase activity by inhibitory iodo compounds (intrathyroidal iodolactones, iodoaldehydes, and iodolipids), affecting the incorporation of iodine into thyroid hormones [1, 4-5]. The reduction of intrathyroidal deiodinase activity related to iodine increase could also contribute to decreased synthesis of these hormones [1].

The acute WCE is usually self-limiting, and normal thyroid function is established considering a physiologic compensatory phenomenon after this protective effect. This could be associated with sodium-iodide symporter (NIS) decreases on the basolateral membrane of thyroid follicular cells due to NIS mRNA and protein decreasing with iodine high levels [6]. Another proposed mechanism is the activation of phosphatidylinositol 3-kinase (PI3K)/protein kinase B (Akt) cascade that also inhibits NIS expression and function in relation to iodine-induced reactive oxygen species production [7]. These mechanisms promote the reduction of intrathyroidal iodine to a critical level which leads to the restart of organification, and the normal synthesis of thyroxine (T4) and triiodothyronine (T3) proceeds [4, 6].

In rare cases, the WCE can lead to hypothyroidism, especially in patients with specific risk factors, such as pre-existing thyroid conditions (Hashimoto’s thyroiditis, Graves’ disease) or surgeries, chronic diseases (cystic fibrosis, thalassemia major, chronic dialysis treatment), use of recombinant interferon-α (rINF-α) therapy, concomitant use of potential goitrogens (amiodarone, lithium, lipofuscin, sulfisoxazole, sulfadiazine), or in those exposed to high levels of iodine for an extended period [1, 4-5, 8]. This occurs due to a failure of a physiological escape phenomenon from WCE and can lead to clinical or subclinical hypothyroidism in a transient form and for a variable period [5, 8].

In radiologic studies, ICM use is a common source of excessive iodine, considering the recommended daily allowance and higher doses may be required in invasive interventional procedures [5]. Some studies describe TD after the use of an ICM [9-12] but the prevalence and clinical significance of it is not yet well characterized [13]. Usually, patients return to a euthyroid state some weeks after ICM exposure, attending to the usual ICM half-life and rapid elimination from the body [5, 13].

In this case, autoimmune thyroid diseases were excluded, and there were no known thyroid diseases in the family. Our patient was diagnosed with HFH, an under-recognized and life-treating rare immunological syndrome characterized by a hyperinflammatory state [14] that could contribute as a predisposing factor to iodine-induced hypothyroidism in a previously euthyroid patient. Because of HFH, she was under physiologic stress, critically ill, and submitted to prolonged hospitalization. Although known alteration of circulating thyroid hormone levels in critical illness [15-16], this patient kept normal thyroid function tests until two weeks after the chemoembolization procedure (Table 1).

Lipidiol® can be used to approach recurrent chylothorax [17-18]. This nonionic low osmolar ICM is an ethiodized oil injection for intralymphatic, intrauterine, and selective hepatic intra-arterial use only [19]. Low osmolar (600-1000 mOsm/kg) contrast agents have few side effects compared to high osmolar but have a higher concentration of iodine [5, 13, 20]. Each millimeter of Lipiodol® contains 480 mg of iodine organically combined with ethyl esters of fatty acids of poppyseed oil [19].

Our patient received an iodine cumulative dose of 4800 mg through thoracic duct chemoembolization by lymphangiography. Attending to iodine’s recommended daily dose of 150 μg and the tolerable upper limit dose of 1100 μg/day for adults [5], she was exposed to an acute excessive dose of iodine. Ethiodized oil reabsorption occurs over a period lasting from a few days to several months or years [19]. This pharmacokinetic property can contribute to the patient’s prolonged exposition to iodine, increasing the risk of failure to escape from the WCE, and inducing hypothyroidism.

The presence of iodides in the urine can be detected as long as ethiodized oil is visible on radiologic images [19]. In this case, urinary iodine excretion was not measured but it is recommended by the last “European Thyroid Association Guidelines for the Management of Iodine-Based Contrast Media-Induced Thyroid Dysfunction” (2021) [13]. A median urinary iodine concentration (UIC) is used as a biomarker of iodine intake, with levels >300 µg/L considered excessive in children and adults (levels >500 µg/L in pregnant women) [1, 13]. No interactions were described with ethiodized oil and patient drugs to treat HFH secondary to granulomatosis disease (corticosteroids and methotrexate) [19]. Therapy for iodine-induced hypothyroidism includes thyroid hormone supplementation and usually patients return to an euthyroid state [13]. Some patients with transitory iodine-induced hypothyroidism may later develop permanent hypothyroidism [5], so it is recommended that patients with a transient TD related to ICM should have a close continuing monitoring of thyroid function [13].

## Conclusions

This case raises awareness of the risk of TD related to ICM, particularly iodine-induced hypothyroidism after the use of ethiodized oil. Although the thyroid has intrinsic regulatory mechanisms when facing high iodine concentrations, hypothyroidism was related to a long-lasting WCE without a physiological escape phenomenon, contributing to a delayed body adaptation to excessive and persistent iodine high levels. This case also emphasizes the importance of thyroid monitoring previously and after the use of ICM, especially in patients with risk factors such as critically ill patients. More studies are needed to better characterize TD related to ICM use, preferably in an international collaborative setting. It is recommended local implementation of guidelines concerning ICM use to prevent and manage adverse events. A causality relation between thyroid dysfunction and the use of an ICM should be reported as an adverse drug reaction to the local services of pharmacovigilance, to improve information to health professionals and patients’ safety.
